# The response of a local health authority to reported cases of salmonellosis in a Portuguese municipality, 2007 to 2011

**DOI:** 10.1186/1756-0500-7-161

**Published:** 2014-03-19

**Authors:** Guilherme Gonçalves, Eduardo Gouveia, Leonie Prasad

**Affiliations:** 1 Instituto de Ciências Biomédicas Abel Salazar, Universidade do Porto, Rua de Jorge Viterbo Ferreira n. 228, 4050-313 Porto, Portugal; 2 Unidade de Saúde Pública, Vila Nova de Famalicão, Portugal

**Keywords:** Salmonellosis, Surveillance, Source, Transmission

## Abstract

**Background:**

Human salmonelloses are statutorily reportable infectious diseases (SRID) in Portugal. Data derived from SRID surveillance systems have been used in international comparisons as well as in studies assessing the sources and modes of transmission of *Salmonella* infections in humans.

**Methods:**

We evaluated a salmonellosis (statutorily reportable) surveillance system in a Portuguese local health authority, consulting routine data available. The main objectives were describing procedures used to investigate and respond to reported cases, and identifying the sources of infection and modes of transmission.

**Results:**

In the five year period from 2007 to 2011, medical doctors reported 58 cases of non-typhoidal salmonellosis to the local health authority. Fifty four reported cases were in hospitalized children (age range 1 – 19 years) and 44 were associated with drinking water from private wells or eating raw egg products, which is consistent with other studies.

**Conclusions:**

This local surveillance system was useful for detecting both isolated cases and outbreaks of salmonellosis and for identifying modes of transmission and sources of infection. It stimulated community health educational activities to prevent future cases. However, further evaluation including economic analysis and an impact assessment is required at both local and national levels.

## Background

Guidelines are available for evaluating public health surveillance systems (PHSS) [1,2]. Statutorily reportable infectious diseases (SRID) systems are examples of PHSS, which are evaluated using different approaches and parameters [3,4]. A PHSS should be systematically evaluated in order to make sure that it fulfills its purpose, using approaches to evaluation that are flexible and individually tailored [1].

Human salmonelloses are statutorily notifiable diseases in a number of developed countries [5], and have been used to evaluate SRID systems [3,4]. Such salmonella-based evaluations have used data from both clinical and laboratory reporting [6,7]. The role of local public health services, in dealing with reported human cases of *Salmonella* infections, has been described and discussed [8,9]. Data derived from SRID surveillance systems have been used in descriptive epidemiological analysis and international comparisons [5] as well as in studies assessing the sources and modes of transmission of *Salmonella* infections in humans [10-13].

Portuguese medical doctors are obliged to report by post (within 48 hours) any confirmed or suspected case of disease, within a list of SRID, to the local health authority (LHA) of the municipality where the case lives [14]. Non-typhoidal salmonellosis (ICD-10 A02) is part of that list. The LHA sends an anonymous paper copy of each individual notification form to the Directorate-General for Health (DGH) [15]. The DGH has issued guidelines addressing the usefulness of reporting SRID, stating that it provides an input for data processing and ensures that reported cases are investigated by the LHA [16], with the explicit purpose of identifying the source of infection and preventing it from spreading [14]. Data availability of SRID should enable epidemiological analysis, useful for both intervention and evaluation [16]. Statistics on Portuguese SRID have been published periodically, the most recent covering the years 2004 to 2008 [17]. Most salmonelloses reported in Portugal were classified as “other” salmonellosis (code A02, in ICD 10) [17] which corresponds to the term “non-typhoidal” salmonellosis as used in the literature [13]; in this paper, the term “salmonellosis” refers to “non-typhoidal” salmonellosis.

We are not aware of published evaluations of salmonellosis surveillance and control, at a local level, in Portugal; neither are there any types of official recommendations on how or when that kind of evaluation should take place. On the other hand, two of the authors who had worked for many years in the LHA, had the impression (anecdotal evidence) that too many reported cases of non-typhoidal salmonellosis in Portugal were waterborne. This would not be expected as more than 83% of the population, in the municipality where this study took place (*Vila Nova de Famalicão*, population 133,000 in 2009), was served by the public water supply system (official data published by the Statistics Portugal [18]). This system undergoes regular quality checks by the relevant water company whose results are sent to the LHA periodically, or as a matter of urgency, if any abnormal laboratory parameter is detected [19]; no problems concerning the quality of the public water supply were identified in the period 2007-2011. The municipality is situated in the North of Portugal, approximately 30 Km from the sea, with both rural and urban industrial areas.

Therefore, we decided to evaluate the local component of the salmonellosis surveillance system, in the municipality where two of the authors were working (GG, EG), and to compare it with other PHSS evaluations using salmonellosis as the example. The main objectives were to:

● Describe the procedures used to investigate reported cases of salmonella;

● Evaluate some qualitative and quantitative aspects of the local PHSS;

● Analyse the sources and modes of human exposure using the routine surveillance data;

● Identify the most likely sources of infection and modes of transmission, which could serve to highlight areas of further investigation.

## Methods

Medical doctors use prepaid mail official forms to notify SRIDs to the LHA [14-16], which include several date fields such as date of onset and date of the signature of the reporting doctor. These forms were personally opened and registered (signed and dated) as “entered” by the five public health doctors, who worked as LHA in this municipality, within the considered period 2007-2011. The forms are in triplicate; the LHA keeps the only one which identifies the patient and sends the other two anonymous copies to the district and national (DGH) hierarchical levels of the Portuguese health authorities. For each reported case of salmonellosis, the LHA created a case-specific file containing a copy of the notification form. When the LHA succeeded in contacting the patient (or the family) a two page written report was produced (in standard format for an epidemiological investigation). When available, additional written documents were added to the individual case file: discharge report from the hospital; laboratory results from samples collected during the investigation; copy of letters sent by the LHA as feed-back information and written notes made during the investigation, which included demographic data and details of the public water supply.

We carefully went through all documents in each case file, and most of the information was computerized. Other information was obtained from hand written reports in the case notes. We used *Epi Info* (*version 3.5.1*) to analyze the data.

Analysis and description of the data focused on the following specific aspects:

● **Operation of the system:** describing how it operates [1], with special emphasis on the procedures concerning investigation and response by the LHA.

● **Quantitative attributes of the surveillance system:** assessing among other parameters, the time between date of onset and receipt of the notification form by the LHA (timeliness).

● **Clinical description of cases:** a description of symptoms and the laboratory tests used to establish the diagnosis and classify the cases.

● **Epidemiological pattern observed:** in addition to the routineclassical description of cases by time, place and people (age and sex), cases were classified as sporadic or as part of an outbreak (defined as 2 or more cases associated in time, place and person).

● **Sources of infection and modes of transmission:** information was available on recognized risk factors like ingestion of raw or undercooked eggs or contaminated water [11,20], use of private wells as sources of drinking water [10,12,20], aquatic recreation [10] contact with pets [13,20], travel outside the municipality of residence in the 3 weeks before onset of disease, etc. Furthermore, we reviewed all information available in each case file, and classified it in terms of the most likely mode of transmission and source of infection as follows: person-to-person fecal-oral transmission [20] or ingestion of contaminated food or water [20]. In some cases, we could not assign one single source, since several were equally likely. In a few cases it was not possible to attribute any source.

● **The response by the local heath authority:** action taken by the LHA, as part of the epidemiological investigation, is summarized, including feed-back given to GPs and reporting medical doctors, and communication with the patients/families, in order to inform them about risk factors and methods for prevention of *Salmonella* infections.

The study was approved by the Ethics Committee of the Portuguese Northern Health Region (*Administração Regional de Saúde do Norte*).

## Results

### Operation of the system

In the five year period from 2007 to 2011, medical doctors reported 58 cases of non-typhoidal salmonellosis to the local health authority (LHA), sending the official pre-paid forms by post. The LHA was successful in contacting 52 of the cases or their relatives (for example the parents of young children) in order to initiate an investigation. Thus, data described here originated from the 58 SRID forms sent by medical doctors, the 52 written reports produced by the LHA after investigation and any additional written information that had been added to each case file.

Although the standard report form used by the LHA promoted the collection of data on specific issues, each LHA was free to conduct the inquiry in the way they found most appropriate, which included: identifying a likely source of infection; taking action to prevent the disease from spreading; taking measures (namely health education activities) to prevent further cases; providing feed-back to the general practitioners (GPs) of the affected patients and involving them in the investigation where applicable; providing feed-back to the medical doctors who reported the cases.

### Identifying the probable source of infection and mode of transmission

For this purpose, the public health doctor in charge of each investigation interviewed the patient or relative either in person or by telephone. An environmental officer visited the homes of 22 cases looking for suspected unsafe water sources like wells or natural springs, taking a water sample at each site that was sent to the laboratory. Twelve of the samples (12/22, 55%) were not considered “adequate for human use” as decreed by Portuguese legislation [19]. Specifically, per 100 ml water, there was either more than one colony of “coliforms” (word used without mentioning specific species) or, in addition to the “coliforms”, there was either *Clostridium perfringens* (any amount), *Escherichia coli* (at more than 80 colonies) or nitrates (any amount). The other organisms recorded as being present were Enterococci (1 colony per 100 ml water) or *Escherichia coli* (1 colony per 100 ml water). Cases or their relatives were informed about the results of all the water samples analyzed.

### Providing feed-back to the family doctors (GPs)

In 36 (69%) of the cases, the LHA informed the patient’s GP about the results of the epidemiological investigation; in some cases, GPs were advised to undertake further laboratory investigations on patients or their relatives to identify asymptomatic carriers of *Salmonella.*

### Providing feedback to the medical doctors who reported the episodes

In 45 (87%) of the cases, the LHA informed the medical doctor who reported the disease on the findings of the epidemiological investigation.

### Some quantitative attributes of the surveillance system

Cases were reported by 33 different medical doctors. One case was reported by a local GP and the remaining 57 by hospital doctors.

For the 58 cases notified, date of onset and date of receipt of the notification form were available. The LHA only knew about most (94%) of the cases more than one week after the date of onset. The time between these two dates (onset and receipt by LHA), varied from 6 to 43 days (see Table 1). This time was calculated as the sum of the time between date of onset and date of notification by the medical doctor and that between the date of notification and date of receipt by the LHA. These two components were similar: the number of days corresponding to the minimum and percentiles 25, 50 and 75 were the same (Table 1). Note that 75% of the cases were known and reported by a medical doctor within one week, but then took several days to arrive at the LHA. This analysis could only be performed in 57 cases because date of notification was missing in one case.

**Table 1:** Distribution of reporting cases of salmonellosis, by time-lag variables (number of days) by extreme values and quartiles

**No of days between specific dates of:**	***n***	**Minimum**	**25%**	**50% (median)**	**75%**	**Maximum**
Onset and reporting by the medical doctor	*57*	2	4	5	7	40
Reporting by the medical doctor and receipt by the LHA	*57*	1	4	5	7	23
Onset and receipt by the LHA	*58*	6	9	12	15	43
Onset and lab confirmation (reported time)	*39*	0	0	1	3	7

Date of laboratory confirmation was available in 39 cases. The time between date of onset and the reported date of laboratory confirmation was within 48 hours in 72% (n = 28) (Table 1).

### Clinical description of cases

Most cases (56/58) were admitted to hospital. The two cases that were not admitted to hospital were children aged one and three years. Symptoms data were available from 52 reports. Fever and diarrhea were the most common (n = 50; 96%), followed by abdominal pain (n = 40; 77%) and vomiting (n = 36; 69%); 78% of cases had a combination of 3 or 4 of these symptoms. Splenomegaly, bradycardia and constipation, were neither reported by the patients nor recorded in hospital reports.

Doctors classified all reported cases as “confirmed on the basis of laboratory data” in the reporting forms but information on the laboratory tests used was just available for 50 of the 52 reports produced by the LHA: *Salmonella* had been cultivated from 46 stool samples and PCR positive results were reported in the other 4 samples.

### Epidemiological pattern observed

The distribution of cases by time (year and month of onset) is displayed in Table 2. In 52% of the cases, date of onset was registered in the trimester May to July, and 31% in the period August to October; very few cases occurred in the remaining months.

**Table 2:** Distribution of the 14 outbreaks identified by the local health authority investigation, by the setting where they occurred, and the number (italic) of cases

	**Total number of cases in the outbreak, Including the initially reported one**				
**Setting where associated cases occurred**	***Two***	***Three***	***Five***	***Eleven***	**TOTAL**
Kindergarten	0	3	0	0	3
Family	4	2	2	0	8
Restaurants (outside the municipality)	0	1	0	1	2
Missing information (*)	1	0	0	0	1
TOTAL	5	6	2	1	14

(*) Patient aged 37 days.

The municipality is divided into 49 small administrative units (called *Freguesias*). No cases were reported in 22 of them, while 24 cases (41%) were resident in the 5 most populated areas (of urban type, containing approximately 34% of the population).

**Figure 1:**
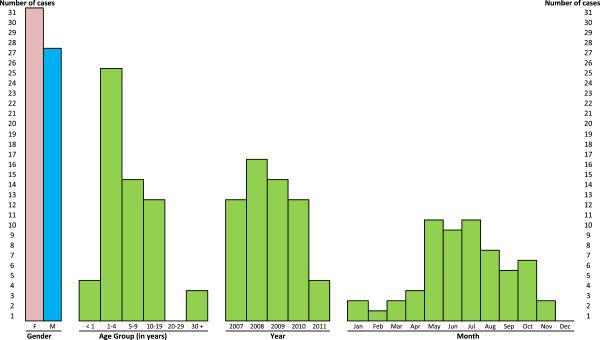
Distribution by gender, age, year and month of occurrence, of the 58 reported cases of salmonellosis.

Age at onset varied from 37 days to 79 years; only four cases occurred in infants (below 1 year of age) and three were adults (Figure 1), aged 31, 74 and 79 years. Cases were more frequent among males (53%).

No secondary cases were identified in 38 of the 52 (73%) epidemiological investigations made by the LHA; we thus assumed that these reported episodes were sporadic. The remaining 14 index cases had further cases linked to them in time, place and/or person and were thus considered to be part of outbreaks (Table 2); three of these 14 had originally been wrongly judged as “sporadic” by the notifying doctor.

Most of the outbreaks (2 or more linked cases) occurred within families involving between two and five cases. Two outbreaks, involving between three and 11 persons (the latter with a suspected source of raw/undercooked egg products) were associated with meals in restaurants. In three of the cases reported in children aged two years, a further two possibly related cases were mentioned to have occurred in kindergarten settings. In the younger case, aged 37 days, the reporting doctor wrote “one additional related case” on the form but no further details were available (“missing information” in Table 2). No details on contact tracing or diagnostic criteria were given, just the numbers of related cases reported by medical doctors, patients or their relatives (Table 2).

### Sources of the infection and mode of transmission

The distribution of cases by known exposure to risk factors is displayed in Table 3. The most common exposure was to raw or undercooked egg products. Seventeen cases had been exposed to unsafe water sources; most of those were private wells apart from two cases, who had also used water from natural springs (one private and another public). Only seven cases had used public swimming pools in the three days before onset of symptoms.

**Table 3:** Exposure to risk factors in the three days before onset of disease, among the 52 cases of salmonellosis infection investigated by the local health authority

**Exposures**	**Number**	**Percentage**
Ingestion of food items		
Raw or undercooked egg products	26	50.0
Other suspected food items	1	2.0
No exposure to suspected food items	19	36.5
Information missing	6	11.5
Water source used in the residence		
Only public water supply	31	59.6
Only well and/or spring	11	21.2
Simultaneous access to safe water and wells/springs	6	11.5
Information missing	4	7.7
Aquatic recreation (swimming pools)		
Yes	7	13.5
No	43	82.7
Information missing	2	3.8

Based on the analysis of all documents in each individual file, we assigned a likely mode of transmission to each case (Table 4). By far the most likely (84.6%) mode of transmission was the ingestion of microorganisms in food or water while person-to-person fecal oral transmission was likely to have occurred in only 2 cases. Travel outside the municipality of residence in the 3 weeks before onset of disease, was reported in 5 cases; in 2 of them, the likely transmission had occurred in restaurants outside the municipality, while in the 3 other cases, travel seemed not to be associated with any particular likely exposure. In 6 cases, we could not ascertain the mode of transmission.

**Table 4:** Likely modes of transmission and sources of infection in 52 cases of reported salmonellosis, investigated

**Cases by likely**	**Number**	**Percentage**
Mode of transmission*		
Ingestion (of contaminated food or/and water)	44	84.6
Person-to-person faecal-oral	2	3.9
Both modes equally likely	0	0.0
Unknown	6	11.5
Source/vehicle ingested (in 44 cases)*		
Raw egg/egg products	22	50.0
Water	18	40.9
Both sources equally likely	4	9.1

*Judgement made by the authors, based on the analysis of all documents in each individual file.

For four of the six cases in whom we could not interview either the patient themselves or a relative, we were still able to assign “ingestion” as the possible mode of transmission because of information written by the reporting doctor in the statutory report.

Among the 44 cases in which “ingestion” was considered the most likely mode of transmission, raw egg/egg products were identified as the most likely source in 22 cases (50%), water from wells and/or springs in 18 cases (41%) and both contaminated food and water in four cases (Table 4). The youngest case (37 days old) was exclusively bottle-fed and the only water source available at home was the private well; in the written report we have no further details except that the LHA has specifically informed the family about the risks of the use of water from sources that may not have been adequately treated.

The two cases classified likely to have been infected by person-to-person fecal oral transmission were children aged two years attending kindergartens, who had not ingested suspected foods or water from unsafe sources, but whose parents mentioned the existence of cases of gastroenteritis in other children from the same kindergarten.

The 6 cases with “unknown” mode of transmission (Table 4) were children aged <1 year (n = 2) 4-5 years (n = 3) and 13 years (n = 1). They were sporadic cases, who had not ingested unsafe foods or water, and there had been no contact with other similar clinical cases.

### The response by the local health authority

The LHA and the environmental officers had produced educational leaflets on the risks of consumption of water from and the ingestion of raw eggs/egg products. They were handed to the patients personally or sent by post. During the epidemiological investigation, the LHA and the environmental officers who visited the patients’ houses also gave verbal advice about the risks of unsafe water and ingestion of raw eggs/egg products. The LHA addressed those issues on the risks of being infected with *Salmonella*, on several occasions, on the local radio and in local newspapers. All kindergartens in the municipality had been inspected at least once by the LHA, as part of a routine public health program; during those visits, both oral and written (leaflets and/or posters) information on the risks of gastroenteritis were given. Whenever the LHA knows of gastroenteritis outbreaks (two or more cases) in kindergartens, these educational activities are reinforced.

## Discussion and conclusions

Almost all reported cases in our municipality were in children (age range 1 – 19 years; 25% aged 0-4 years) who had been hospitalized due to clinical syndromes with moderate severity. Within the European Union (EU), there is usually a peak of cases in the summer months [21], and our findings are similar in that 47% (27/58) of cases occurred between June and August. In developed countries, it has been estimated that only about 1% of clinical cases are reported [20] and we assume that the situation is similar in Portugal though we did not have data to sensitivity [4].

Our observed timeliness from the date of onset to receipt and acknowledgement of the report by the LHA (range 6 – 43 days) were slightly longer than those reported in a USA study where the state-specific reporting range was 2 – 44 days from date of onset to public health verification or investigation of health event report [3]. However, the timeliness analysis made was not directly comparable since our end point measured was receipt of the report by the LHA rather than verification or investigation of the health event report. But like the USA study [3] the low percentage of time values below one incubation period (defined as 1.5 days in Jajosky et al [3]), limits effective immediate interventions. In this study, epidemiological characteristics of cases and timeliness issues possibly prevented the LHA from interrupting chains of transmission; anyway, these are not generally the kind of situations where a LHA could prevent the disease from spreading. Nevertheless, this SRID system allowed the LHA to identify behavioral risks and intervene on a case by case basis (people affected should be highly motivated) to avoid future infections with *Salmonella,* or other agents with similar modes of transmission.

We have not assessed the impact of giving feedback to reporting doctors and GPs, but that procedure has been identified by other authors as potentially useful for implementing contact screening [8], which might be very costly [8], and for improving completeness and timeliness of notification [22]. On the other hand local public health services have sometimes been criticized for their “lack of communication” with GPs and hospitals [9].

This was a descriptive study, in which measures of association could not be estimated. Nevertheless, based on the information available we observed that most cases had been exposed to avoidable risks like water that may not have been adequately treated such as private wells and natural springs and/or raw eggs. The diagnosis of *Salmonella* infections among people using private wells as sources of drinking water is consistent with findings from other studies [10,12]. In our study it was observed that people eating raw or undercooked eggs, could get clinically relevant *Salmonella* infections, which is also consistent with studies elsewhere [11]. But given the availability of safe water and food in this community, such exposures should be considered unacceptable. Theoretically, this disease can be easily prevented. It was evident that a number of residents in this municipality were not aware that their behaviors could increase their risk of acquiring *Salmonella* or other enteric infections. Person-to-person contact was a rare mode of transmission in this study (two cases), but has been reported more frequently in certain settings, for example, in day care centres [13].

The LHA has been involved in health education activities to prevent future cases of *Salmonella* infection, by giving personalized advice during the investigation, as well as routinely through planned health education activities in school settings and the local media. However, we are not aware of any local studies to assess the effectiveness of such routine health educational activities. There is not always a direct relationship between knowledge and health behaviours [23]. Public health services should focus on evidence-based [23,24] activities to prevent future cases of *Salmonella* infection (and other diseases with similar modes of transmission and sources of infection). Meanwhile, educational interventions similar to those used by this LHA have been recommended and used in other countries, like visiting families and schools [9,24] to educate parents and caretakers about the risk of *Salmonella* transmission in children [13], and using local media [9] to convey health educational messages. Interventions in schools have also been reported to be useful for increasing awareness of the dangers of using, and thus reducing the use of, unsafe water sources [24].

In conclusion, the PHSS in this municipality was useful for detecting cases of salmonellosis and for identifying likely modes of transmission and sources of infection. It stimulated community health educational activities to prevent future cases. However, further, more detailed, evaluation of the PHSS, including economic analyses and impact assessments, is required at both local and national levels, which will also be useful when comparing our PHSS to those of other countries.

## Competing interests

The authors declare that they have no competing interests.

## Authors’ contributions

The field work was done in the municipality of Vila Nova de Famalicão, in the North of Portugal, where GG and EG had been working has Local Health Authorities (LHA) for years; EG still works there; GG was the Head of the LHAs but has moved full time to the University of Porto in 2009. GG conceived and designed the study. With the other public health doctors working as LHAs, GG and EG were actively involved in studying some of the reported cases. EG organized the individual data files. GG and LP performed the analysis and wrote the manuscript. All authors read and approved the final manuscript.

## References

[B1] Klaucke DN Teutsch SM, Churchill RE Evaluating Public Health Surveillance Principles and Practice of Public Health Surveillance 1994 New York: Oxford University Press 158 174

[B2] CDC Guidelines Working Group Updated guidelines for evaluating public health surveillance systems Morb Mortal Wkly Rep 2001 50 1 35 18634202

[B3] Jajosky RA Groseclose SL Evaluation of reporting timeliness of public health surveillance systems for infectious diseases BMC Public Health 2004 4 29 10.1186/1471-2458-4-29 15274746 PMC509250

[B4] Jansson A Arneborn M Ekdahl K Sensitivity of the Swedish statutory surveillance system for communicable diseases 1998-2002, assessed by the capture-recapture method Epidemiol Infect 2005 133 401 407 10.1017/S0950268804003632 15962546 PMC2870263

[B5] Jong B Ekdahl K The comparative burden of salmonellosis in the European Union member states, associated and candidate countries BMC Public Health 2006 6 4 10.1186/1471-2458-6-4 16403230 PMC1352352

[B6] Swaminathan B Barrett TJ Fields P Surveillance for human Salmonella infections in the United States J AOAC Int 2006 89 553 559 16640306

[B7] Takahashi T Koehler J Swenson P Duchin J Evaluation of a public health Salmonella surveillance system in King County, Washington Am J Infect Control 2004 32 7 11 10.1016/j.ajic.2003.06.003 14755228

[B8] Thomas HL Addiman S Mellanby A Evaluation of the effectiveness and efficiency of the public health management of cases of infection due to Salmonella typhi/paratyphi in North East London Public Health 2006 120 1188 1193 10.1016/j.puhe.2006.06.013 17010396

[B9] Diack L Smith DF Professional strategies of medical officers of health in the post-war period–1: ‘innovative traditionalism’: the case of Dr Ian MacQueen, MOH for Aberdeen 1952–1974, a ‘bull-dog’ with the ‘hide of a rhinoceros’ J Public Health Med 2002 24 123 129 10.1093/pubmed/24.2.123 12141581

[B10] Denno DM Keene WE Hutter CM Koepsell JK Patnode M Flodin-Hursh D Stewart LK Duchin JS Rasmussen L Jones R Tarr PI Tri-county comprehensive assessment of risk factors for sporadic reportable bacterial enteric infection in children J Infect Dis 2009 199 467 476 10.1086/596555 19281302 PMC3693595

[B11] Domingues AR Pires SM Halasa T Hald T Source attribution of human salmonellosis using a meta-analysis of case-control studies of sporadic infections Epidemiol Infect 2012 140 959 969 10.1017/S0950268811002172 22152439

[B12] Uhlmann S Galanis E Takaro T Mak S Gustafson L Embree G Bellack N Corbett K Isaac-Renton J Where’s the pump? Associating sporadic enteric disease with drinking water using a geographic information system, in British Columbia, Canada, 1996-2005 J Water Health 2009 7 692 698 10.2166/wh.2009.108 19590137

[B13] Younus M Wilkins MJ Davies HD Rahbari MH Funk J Nguyen C Siddiqi AA Cho S Saeed M Case-control study of disease determinants for non-typhoidal Salmonella infections among Michigan children BMC Res Notes 2010 3 105 10.1186/1756-0500-3-105 20398398 PMC2862038

[B14] Portugal. Presidency of the Republic Lei 2:036 Diário do Governo, Volume I Série – Número 175 1949

[B15] Portugal. Directorate-General for Health Doenças transmissíveis de Declaração Obrigatória. Extinção das Sub-Regiões de Saúde e alteração do circuito de Informação Circular Normativa Nº 05/DSEES/DE, 06/04/2009

[B16] Portugal. Directorate-General for Health Doenças de Declaração Obrigatória Circular Normativa N° 9/80, 09/05/1980

[B17] Portugal. Directorate-General for Health. Direcção de Serviços de Epidemiologia e Estatísticas da Saúde. Divisão de Epidemiologia – Lisboa: DGS, 2010 Doenças de Declaração Obrigatória, 2004-2008 Estatísticas de Saúde [http://www.portaldasaude.pt/NR/rdonlyres/1FE557EF-97D1-4C6F-9C40-36529B45538C/0/DoencasDeclaracaoObrigatoria_DGS.pdf]

[B18] Portugal. Instituto Nacional de Estatística/Statistics Portugal Estatísticas territoriais. Vila Nova de Famalicão [http://www.ine.pt/bddXplorer/htdocs/printable.jsp?id=c1c0071b30d87bf5852ba85a4fa4b1d3f58eb5e555f5_47876&lingua_cd=PT]

[B19] Portugal. Ministry of Environment Decreto-Lei n.° 306/2007 Diário da República, Volume 1.ª série — N.° 164

[B20] Heymann DL Control of Communicable Diseases Manual 2004 18 Washington DC: American Public Health Association 470 471

[B21] European Centre for Disease Prevention and Control (ECDC) Annual Epidemiological Report 2011. Reporting on 2009 Surveillance Data and 2010 Epidemic Intelligence Data 2011 Stockholm: ECDC

[B22] Allen CJ Ferson MJ Notification of infectious diseases by general practitioners: a quantitative and qualitative study Med J Aust 2000 172 325 328 10844919 10.5694/j.1326-5377.2000.tb123979.x

[B23] Nesbitt A Majowicz S Finley R Marshall B Pollari F Sargeant J Ribble C Wilson J Sittler N High-risk food consumption and food safety practices in a Canadian community J Food Prot 2009 72 2575 2586 20003742 10.4315/0362-028x-72.12.2575

[B24] Yuan L Manderson L Temongko MS Wei W Aiguo P The impact of educational videotapes on water contact behaviour of primary school students in the Dongting Lakes region, China Trop Med Int Health 2000 5 538 544 10.1046/j.1365-3156.2000.00602.x 10995095

